# The Impact of Anthropometric Characteristics and Motor Abilities on Match Running Performance (MRP) in Elite Soccer Players: A Multiple Regression Approach

**DOI:** 10.5114/jhk/207525

**Published:** 2025-11-19

**Authors:** Radivoje Radaković, Borko Katanić, Mima Stanković, Igor Jelaska, Goran Jelaska

**Affiliations:** 1 Institute of Information Technologies, University of Kragujevac, Kragujevac, Serbia.; 1 Bioengineering Research and Development Center, Kragujevac, Serbia.; 3 Montenergin Sports Academy, Podgorica, Montenegro.; 4 Faculty of Sport and Physical Education, University of Niš, Niš, Serbia.; 5 Faculty of Kinesiology, University of Split, Split, Croatia.; 6 Virovitica County hospital, Virovitica, Croatia.

**Keywords:** professional football, morphological characteristics, body composition, motor abilities, sprint abilities, field tests

## Abstract

The aim of the study was to examine the impact of anthropometric characteristics and motor abilities on match running performance (MRP) in elite soccer players. For this purpose, a sample of 41 professional soccer players was evaluated considering five anthropometric characteristics, 11 motor abilities (including sprint and acceleration performance, agility, power, and endurance) as regressors, and six variables describing MRP (time spent in running at < 8 km/h, 8–15 km/h, 15.1–19 km/h, 19.1–23 km/h, > 23 km/h and total time spent in running) as criteria. Regression analysis identified significant regression models (p < 0.05) predicting 8.1–15 km/h, 19.1–23 km/h, and total time spent in running variables for both sets of regressors, with morphology explaining 29.6%, 29.8%, and 37.4%, and motor abilities explaining 59.6%, 65.9% and 68.7% of criterion variables, respectively. It can be concluded that body composition variables such as muscle and fat percentages are associated with jogging, high-speed running, and total distance covered, whilst sprinting variables at 10, 20, and 30 m, the 10/20 index, and the CMJ are associated with jogging and total distance covered. Also, the squat jump is associated with jogging, while shuttle run test performance is associated with high-speed running only. Results provide deep insight into the structure of relationships between MRP and both morphological and motor variables, which are essential to structure advanced training systems focused on optimization of MRP in elite soccer players.

## Introduction

Competitive soccer is a highly intermittent activity that requires professional players to simultaneously perform multiple tasks, primarily demanding agility, strength, speed, balance, stability, flexibility, and endurance (Drust et al., 2007). Consequently, preparing players for real-life match scenarios in elite competitions necessitates the integration of scientific knowledge into practical training settings (Asimakidis et al., 2024a; Čaprić et al., 2022; Filter et al., 2023). Achieving excellence in modern soccer requires an optimal and synergistic balance of technical, tactical, physical, and psychological performance (Asimakidis et al., 2024b; Versic et al., 2022). The interdependence of these components underscores the need for comprehensive preparation at both the individual and team levels (Stanković et al., 2022).

Research consistently highlights the importance of optimal anthropometric characteristics in providing a competitive advantage during matches (Ré et al., 2014). Increasing lean body mass is recognized as one of the most critical factors for optimizing sports performance (Stankovic et al., 2023). While various anthropometric variables are often associated with advanced biological maturity, research has shown that higher-level players tend to exhibit greater body height and fat-free mass (Waldron and Worsfold, 2010) Additionally, soccer players with lower fat mass in favor of lean mass may demonstrate superior performance in change of direction (COD) speed, high-intensity intermittent running, vertical jumps, and sprinting (Hazir, 2010). Furthermore, the body fat percentage and fat-free mass are linked to maximal running capacity and vertical jump performance (Esco et al., 2018), as well as repeated sprint ability (Campa et al., 2019). Research also suggests that an excess of adipose tissue and a deficiency in muscle mass may negatively impact athletic performance (Bongiovanni et al., 2021).

With recent advancements in the technical and tactical aspects of soccer, performance analysis has become essential for evaluating players’ performance (Ehrmann et al., 2016; Kubayi and Toriola, 2020; Morgans et al., 2023). Additionally, field-based evaluations are increasingly used to assess endurance, muscular strength, and sprinting performance under conditions that closely replicate match-play demands (Zagatto et al., 2016). GPS technology further enhances performance analysis by enabling the collection of detailed data on players' running performance, including total distance traveled, distance covered at various speeds, and the number of accelerations and decelerations (Aquino et al., 2020). Another critical aspect of scientific and practical soccer performance analysis is the use of game performance indicators. These indicators consist of selected and structured variables that define specific components of performance and contribute to athletic success (Sarmento et al., 2014)

There are inconsistencies in the literature regarding the relationship between anthropometric and physical performance characteristics and match-play running demands. Research indicates a negative association between subscapular skinfold thickness and total distance covered, high-intensity running, and sprinting in youth athletes (Buchheit and Mendez-Villanueva, 2014). Furthermore, studies have found limited relationships between match running performance (MRP) and factors such as age, maturation status, and body size in elite youth players. However, for both midfielders and forwards, most analyzed variables exhibit significant associations with high-intensity MRP, including the frequency of both single and repeated high-intensity actions, which correlate with maturation status and body size. Additionally, research has identified links among body mass, muscle mass, and the total distance covered during soccer matches (Rienzi et al., 2000). Moreover, anthropometric data suggest a lack of correlation between match running demands at the junior and senior levels (Aquino et al., 2020). However, match performance has been associated with various physiological and field-performance indicators in both professional and youth players, including blood lactate concentration, running duration, and VO_2max_. Several field-based variables have also been linked to in-game running performance, with studies reporting moderate correlations between running profiles, change-of-direction ability, and anaerobic sprint performance (Aquino et al., 2017; Goncalves et al., 2021; Rampinini et al., 2006). Players who achieved higher scores in the Yo-Yo Intermittent Recovery Test Level 1 (Yo-Yo IR1) demonstrated greater total distance covered and engaged more in higher-intensity running during matches (Castagna et al., 2009). Conversely, some studies suggest that various physical fitness assessments, including jump performance, repeated sprint ability (RSA), sprint time, and aerobic capacity, are not significantly correlated with total running performance during soccer matches (Aquino et al., 2017; Rago et al., 2018).

Some authors discussed that the link between physical fitness and MRP was not as strong as previously thought (Buchheit et al., 2010). This was due to the fact that correlation coefficients are partially dependent on the playing position. In summary, inconsistencies in the literature regarding the associations between MRP and both the motor abilities and morphological characteristics highlight the need for further research. Therefore, the aim of this study was to examine the impact of anthropometric characteristics and motor abilities on match running performance variables in elite soccer players.

## Methods

### Participants

The study sample consisted of professional soccer players (n = 41; age: 23.20 ± 3.40 years), all competing in the Super League, the highest tier of national competition. Only players aged 18 to 35 years with a minimum of six years of competitive experience were included. Participants were required to be free from any recent musculoskeletal injuries or illnesses at the time of the evaluations. Each participant provided written informed consent and voluntarily participated in the study after being thoroughly briefed on its objectives, potential benefits, and risks. All data were anonymized, and the study adhered to the ethical principles outlined in the Helsinki Declaration (Association, 2013). Ethical approval was obtained from the Ethics Committee of the Faculty of Medical Sciences, University of Kragujevac, Kragujevac, Serbia (protocol code: 01-15731; approval date: 29 December 2021).

### Procedures

Anthropometric assessments included four variables: body height, body mass, the body fat percentage, and the muscle mass percentage. Before the measurements, participants completed a standardized warm-up consisting of 8 min of moderate jogging, 5 min of static stretching, and 2 min of short bursts of high-intensity running. This warm-up protocol has been implemented in similar previous studies involving athletes, following the same sequence of activities and a comparable time frame (Arslan et al., 2022; Dupont et al., 2004; Ouerghi et al., 2017). Following the warm-up, participants underwent a series of motor tests covering 11 variables. These tests included linear sprints over 10 m, 20 m, and 30 m, from which acceleration indices for 10/20 and 10/30 were derived. Additionally, agility performance was assessed using a zig-zag agility test, performed both with and without a ball. Participants also completed countermovement jumps (CMJs), squat jumps (SJs) without an arm swing, and a shuttle run test to evaluate endurance. All tests were conducted on a soccer field during March 2022, in the morning at approximately 11 a.m., following standardized order: the CMJ, the SJ, 10-m, 20-m, and 30-m sprints, the zig-zag test without a ball, the zig-zag test with a ball, and finally, the shuttle run test. Match running performance was evaluated during official competitive matches. For each player, the total distance covered over the 90-min match was recorded and categorized into five speed zones, along with the total distance covered.

### Anthropometric Characteristics

Anthropometric assessments were conducted following the guidelines of the International Biological Program (Eston and Reilly, 2009). Body height was measured using a GPM anthropometer (GPM, Zurich, Switzerland) with accuracy of ±0.1 cm, while body mass was assessed using a Tefal 6010 scale (Rumilly, Haute-Savoie, France) with accuracy of ±0.1 kg. The body mass index (BMI) was calculated using the formula: BMI = BM (kg) / BH^2^ (m^2^), where BM represented body mass and BH denoted body height. The body fat percentage and muscle mass were indirectly assessed using the bioelectrical impedance analysis (BIA) method, employing the Omron BF 300 device (Omron Corp, Kyoto, Japan). The Omron BF 300 has been shown to be a valid device for assessing body composition (Martín Moreno et al., 2001). For the purpose of body composition analysis, participants were advised to refrain from consuming food or drink for at least 2 to 4 h prior to the measurement in order to avoid hydration variations. Physical activity was also avoided for at least 6 to 12 h before the test, as it could affect the distribution of water in the body. Measurements were taken barefoot and without metal objects, such as jewelry, to ensure accurate electrical conductivity (Pribylet al., 2011). The percentage values for body fat and muscle mass were obtained directly from the device display, with accuracy of ±0.1% (Eston and Reilly, 2009).

### Linear Sprint Performance

The sprint tests required participants to cover distances of 10, 20, and 30 m in the shortest possible time. Sprint performance in soccer has been measured in this way in previous studies, including sprints over 10–20 m (Mirkov et al., 2008; Reichert et al., 2025; Sonesson et al., 2021) and 10–30 m (Parpa et al., 2024; Walker and Turner, 2009). These distances were chosen because, in professional soccer, the mean distance and duration of sprint runs are relatively short—sprinting distances rarely exceed 20 m and typically last no longer than 4 s (Di Salvo et al., 2006). Furthermore, Andrzejewski et al. (2013) reported that 90% of sprints performed by professional soccer players were shorter than 5 s.

Timing was measured using electronic timing gates (Witty, Microgate, Bolzano, Italy) positioned 1 m above the ground at the start line, as well as at the 10-m, 20-m, and 30-m (finish) lines. The test commenced when the participant disrupted the beam of a photoelectric cell at the start and concluded upon breaking the beam at the designated target point. Each participant performed two sprints, with a two-min rest interval between trials. This rest between the two sprint attempts was chosen because it allowed for adequate recovery of the ATP-PCr energy system, which is crucial for performing maximum short-duration efforts. Research shows that for sprint efforts of similar duration and intensity, rest periods of 1.5 to 3 min enable nearly complete recovery of speed and strength, without significant accumulation of fatigue between attempts (Glaister, 2005). Split times for 10 m, 20 m, and 30 m were recorded for each attempt, and the better result was retained for further analysis. Additionally, acceleration indices were calculated for the 20-m distance relative to 10 m and for the 30-m distance relative to 10 m.

### Agility Tests

Agility was assessed using the zig-zag test, performed both without a ball and with a ball. During the test, participants were instructed to cover four segments of 5 m each at maximum speed. These segments were set at an angle of 100° relative to each other. The test evaluated the time required to complete the course, from which speed and acceleration were subsequently calculated. Additionally, the Skill Index (SI) was determined as the quotient of the time recorded in the zig-zag test without a ball and the time recorded in the zig-zag test with a ball. These Zig-zag tests with and without the ball have been used in numerous studies with soccer players (Mirkov et al., 2008; Walker and Turner, 2009).

### Jump Performance

Lower extremity power was assessed using the Squat Jump (SJ) and Countermovement Jump (CMJ) tests. All jumps were performed on a photocell mat (Optojump, Microgate, Bolzano, Italy). For both tests, participants were given instructions for the proper execution of the tests—maintaining a straight back, placing hands on the hips, trunk fixation, and ensuring a uniform squat depth (e.g., up to 90° knee flexion)—which contributed to reducing variability between trials and participants. Such standardization enhanced the repeatability and validity of the test, as confirmed by recommendations from the literature (Cormie et al., 2009; Markovic et al., 2004). The SJ test was executed from a squatting position with approximately 90° knee flexion, without an arm swing. In contrast, the CMJ was performed from a similar squatting position but included a dynamic countermovement, utilizing a strong arm and body swing to maximize jump height. Both tests measured the maximum vertical jump height in centimeters. Test-retest reliability of the Optojump system was excellent (ICCs ranging from 0.982 to 0.989), and also the intraclass correlation coefficients (ICCs) for validity were very high (0.997–0.998) (Glatthorn et al., 2011).

### Shuttle Run Test

Endurance was assessed using the shuttle run test, which correlates with movement performance of soccer players during matches (Castagna et al., 2010; Lemmink et al., 2004). In this test, participants were required to run between two cones placed 20 m apart, with the running pace determined by an auditory signal from an audio recording of the protocol. The initial running speed was set at 8 km/h, with the speed increasing by 0.5 km/h every minute. If a participant failed to reach the starting marker within the specified time interval for the first time, they received a warning. If the delay occurred again, the test was terminated. The primary measure of the test was the total distance covered by the participant, in meters. This test was conducted according to the existing protocol (Ramsbottom et al., 1988), which is commonly used in soccer research (Lesinski et al., 2017; Nassis et al., 2010; Perroni et al., 2024).

### Match Running Performance (MRP)

The BioIRC Tracking Motion system (BioIRC, Kragujevac, Serbia) was used to record soccer matches. This system utilizes two identical Sony NEX-VG10 video cameras (Sony, Tokyo, Japan) in full HD resolution, along with a high-speed control camera. The cameras were installed in fixed positions on the roof of the stadium. The Tracking Motion BioIRC software system has demonstrated high internal and external validity during competitive soccer matches (Radaković et al., 2015, 2020). Also, it has been employed in previous studies (Radaković et al., 2024a, 2024b). The algorithm for tracking the Player Movement Profile is based on measuring the similarity in the statistical color distribution of objects. Video analysis involves monitoring players’ performances across the field by processing video recordings of each half alternately. The processing speed on the computer was approximately four frames per second. Match videos were compressed using the K-SVID codec in the MOV format, with a frame rate of 30 frames per second.

Match running performance (MRP) for players was determined using the BioIRC analysis model, which followed established standards for profiling movement intensities in relation to physical and physiological variables. Five categories of movement intensities during the match were defined, and a sixth variable, representing overall player movement, was calculated based on the measured variables (Di Salvo et al., 2013; Lago-Penas et al., 2009; Radaković et al., 2024b).

### Variables

The independent variables in this study can be categorized into three groups: anthropometric characteristics, motor abilities and MRP. A total of 22 variables were divided into three groups, with five variables representing anthropometric characteristics, 11 variables representing motor abilities and six variables representing MRP. The first group, i.e., anthropometric characteristics, included: body height (BH), body mass (BM), body mass index (BMI), fat mass percentage (%FM), and muscle mass percentage (%MM). The second group, i.e., motor abilities, comprised: 10–30 m sprints (10–30 m), acceleration indexes (10 m/20 m, 10 m/30 m), zig-zag agility with and without a ball, a squat jump (SJ) and a countermovement jump (CMJ), and the shuttle run test (Table 1). Finally, MRP variables included sums of distances covered in movement (in meters) within specific speed ranges: walking (< 8 km/h), jogging (8–15 km/h), running (15.1–19 km/h), high-speed running (19.1–23 km/h), sprinting (> 23 km/h), and the total distance covered (Total). Table 1 provides an overview of variables and their abbreviations.

**Table 1:** Anthropometric characteristics, motor abilities and running performance variables.

No.	Variables	Abbreviation
1.	Body height	BH
2.	Body mass	BM
3.	Body mass index	BMI
4.	Fat mass percentage	%FM
5.	Muscle mass percentage	%MM
6.	10-m sprint	10 m
7.	20-m sprint	20 m
8.	30-m sprint	30 m
9.	Zig-zag agility	Zig-zag
10.	Zig-zag agility with a ball	Zig-zag Ball
11.	Index acceleration 10/20 m	Index 10/20
12.	Index acceleration 10/30 m	Index10/30
13.	Index agility zig-zag	Index zig-zag
14.	Squat Jump	SJ
15.	Countermovement jump	CMJ
16.	Shuttle run	Shuttle
17.	Walking (from 0 to 8 km/h)	< 8 km/h
18.	Jogging (from 8 to 15 km/h)	8–15 km/h
19.	Running (from 15.1 to 19 km/h)	15.1–19 km/h
20.	High-speed running (from 19.1 to 23 km/h)	19.1–23 km/h
21.	Sprinting (over 23 km/h)	> 23 km/h
22.	Total distance covered	Total

### Statistical Analysis

All data were presented as mean ± standard deviation, minimum, maximum, and range. Relationships among the predictors and the criterion variables were assessed using series of 12 multiple regressions. For each of 6 criterion variables (< 8 km/h, 8–15 km/h, 15.1–19 km/h, 19.1–23 km/h, > 23 km/h, Total) two sets of regressors were associated to it: morphology (BH, BM, BMI, %FM, %MM) and motor abilities (10 m, 20 m, 30 m, Zig-zag, Zig-zag Ball, Index 10/20, Index 10/30, Index zig-zag, SJ, CMJ, Shuttle). Standardized coefficients (Beta) along with associated significance levels (*p*) were calculated together with the coefficient of multiple correlation (R) and multiple determination (R2); Type I error was set at α < 0.05. Data analysis was conducted using IBM SPSS Statistics 26 software (Statistical Package for Social Sciences, v26.0, SPSS Inc., Chicago, IL, USA).

## Results

The results presented in Table 2 provide the descriptive characteristics of the studied professional soccer players. The average body height and mass of players were 182.00 cm and 76.86 kg, respectively. Additionally, players demonstrated a low body fat percentage of 9.55%.

**Table 2:** Descriptive results of anthropometric, motor, and match-running performance (MRP) variables of elite soccer players.

Variables	Mean ± SD	Min	Max	Range
BH (cm)	182.00 ± 5.15	172.00	192.00	20.00
BM (kg)	76.86 ± 6.06	63.00	88.00	25.00
BMI (kg/m^2^)	23.22 ± 1.25	19.90	25.50	5.60
%FM (%)	9.55 ± 2.45	3.70	14.10	10.40
%MM (%)	43.42 ± 1.72	40.10	46.50	6.40
10 m (s)	1.62 ± 0.08	1.45	1.76	0.31
20 m (s)	2.92 ± 0.12	2.72	3.20	0.48
30 m (s)	4.05 ± 0.15	3.77	4.45	0.68
Zig-zag (s)	6.72 ± 0.58	5.86	7.56	1.70
Zig-zag B (s)	8.17 ± 0.86	6.93	9.87	2.94
Index 10/20 m	0.55 ± 0.03	0.50	0.60	0.10
Index 10/30 m	0.40 ± 0.02	0.36	0.49	0.13
Index zig-zag	0.83 ± 0.04	0.73	0.92	0.18
SJ (cm)	42.00 ± 5.82	21.50	51.40	29.90
CMJ (cm)	53.44 ± 7.90	41.10	70.20	29.10
Shuttle (m)	2476.59 ± 235.71	2020.00	2860.00	840.00
< 8 km/h (m)	4522.56 ± 450.43	3301.36	5243.99	1942.63
8–15 km/h (m)	4193.87 ± 831.19	2580.46	5850.53	3270.07
15.1–19 km/h (m)	983.55 ± 247.63	502.00	1393.53	891.53
19.1–23 km/h (m)	611.45 ± 154.36	319.33	885.60	566.27
> 23 km/h (m)	488.38 ± 144.99	261.26	893.85	632.59
Total (m)	10799.79 ± 1143.70	8429.52	12602.25	4172.73

Note: mean value ± standard deviation (Mean ± SD), minimal result (Min), maximal result (Max), data range (Range)

Regarding motor abilities, the sprint times for the 10-m, 20-m, and 30-m distances were 1.62 s, 2.92 s, and 4.05 s, respectively. The results for the SJ and CMJ tests equaled 42.00 cm and 53.44 cm, respectively. In the shuttle run test, players covered an average distance of 2476.59 m.

In terms of movement performance, the total distance covered was 10.799 m, while the players covered an average of 611 m during high-intensity running and 488 m while sprinting.

The results of the multiple regression analysis (Table 3) show that the model with anthropometric variables explained a total of 37.4% of the variance in the dependent variable “total distance covered” at a significant level (R^2^ = 0.374). The greatest contributions to the model came from %FM (−0.592) and %MM (−0.543), with the negative beta coefficients indicating that higher values of body fat content and muscle mass predicted a lower total distance covered. In the speed zone of 8.1–15 km/h, the model with anthropometric variables explained 29.6% of the variance (R^2^ = 0.296), with significant contributions from both %FM (−0.487) and %MM (−0.519). Similarly, in the 19.1–23 km/h zone, the model explained 29.8% of the variance (R^2^ = 0.298), with a significant negative influence from %FM (−0.397) and %MM (−0.386). In the other speed zones, the explained variance was lower, and the models were not significant.

**Table 3:** Multiple regression analysis—beta coefficients and variables of the regression model.

Variables	< 8 km/h	8.1−15 km/h	15.1−19 km/h	19.1−23 km/h	> 23 km/h	Total
BH	1.260	0.473	−0.657	0.902	−1.003	0.692
BW	−1.817	−0.908	0.632	−1.548	1.196	−1.296
BMI	1.177	0.847	−0.187	1.416	−0.737	1.137
%FM	−0.364	−0.487*	−0.201	−0.397*	0.018	−0.592*
%MM	−0.061	−0.519*	−0.388	−0.386*	−0.046	−0.543*
R	0.394	0.544	0.482	0.546	0.219	0.612
R^2^	0.156	0.296	0.232	0.298	0.048	0.374
p	0.290	0.026	0.087	0.024	0.878	0.004
10 m	−0.107	−6.205*	−6.841	−4.049	−1.999	−6.833*
2 0m	0.299	4.345	4.838	2.661	1.786	4.909*
30 m	−0.230	0.579*	0.498	0.306	−0.442	0.423*
Zig-zag	5.943	−0.128	0.628	−0.987	1.524	2.444
Zig-zag B	−6.641	0.786	−0.687	1.686	−2.050	−2.225
Index 10/20	0.010	5.587*	6.200	3.775	2.017	6.172*
Index 10/30	0.013	−0.053	0.125	0.024	−0.111	−0.017
Index zig-zag	−3.263	0.418	−0.318	0.738	−1.023	−1.080
SJ	−0.389	0.460*	0.346	0.090	−0.045	0.262
CMJ	0.404	−0.466*	−0.548	−0.220	−0.305	−0.367*
Shuttle	0.170	−0.141	−0.240	−0.297*	0.005	−0.127
R	0.638	0.772	0.628	0.812	0.411	0.829
R^2^	0.407	0.596	0.394	0.659	0.169	0.687
P	0.099	0.002	0.119	0.000	0.863	0.000

Note: R: Coefficient of multiple correlation; R2: coefficient of determination; p: statistical significance; * denotes statistical significance of Beta coefficient (p < 0.05)

**Figure 1:**
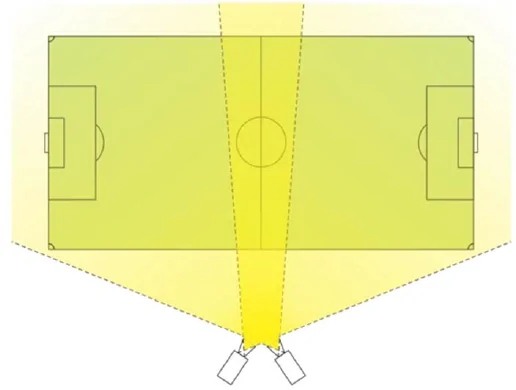
Positions of ultra-high-speed cameras during match recording.

**Figure 2:**
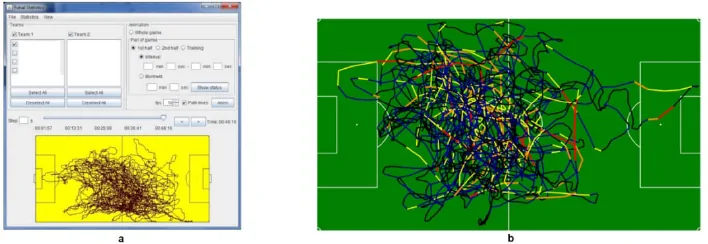
(a) Overview of the BioIRC Tracking Motion software interface; (b) visual representation of a single player's trajectory throughout halftime, segmented into classifications reflecting varying movement intensities.

When motor abilities were included in the analysis, the model significantly explained 68.7% of the variance in the total distance covered (R^2^ = 0.687). The greatest contributions were from the 10-m sprint (−6.833) and the 10/20 index (6.172), indicating that better sprint performance strongly contributed to a greater total distance covered. In the speed zone of 19.1–23 km/h, the model with motor abilities explained 65.9% of the variance (R^2^ = 0.659), with a significant contribution from the shuttle test (−0.297), indicating that better aerobic endurance contributed to a greater distance covered in this zone. In the 8.1–15 km/h zone, the model explained 59.6% of the variance (R^2^ = 0.596), with significant contributions from the 10-m sprint (−6.205), the squat jump (SJ) (0.460), and the 10/20 index (5.587).

Significant models were not found for the lowest (< 8 km/h) and highest (> 23 km/h) speed zones, where the R^2^ values were low (0.156 and 0.048 for the anthropometric variables, and 0.407 and 0.169 for the motor characteristics), and the beta coefficients did not reach statistical significance.

## Discussion

This study aimed to examine the association between anthropometric characteristics and motor abilities with match running performance (MRP) variables in elite soccer players. The main findings of the study, based on multiple regression analysis, were as follows: (i) body composition variables such as muscle and fat percentages were associated with jogging, high-speed running, and total distance covered; (ii) sprinting variables at 10, 20, and 30 m, the 10/20 index, and the CMJ were related to jogging and total distance, while the SJ was only associated with jogging; (iii) performance on the shuttle run test was associated with high-speed running.

To accurately analyze soccer players' performance, laboratory and field measurements are necessary, along with competitive performance data obtained from direct software measures of the player's motions during the soccer match (Radaković al., 2024b). Our results confirmed that the sample used consisted of elite players considering their morphological characteristics (Gardasevic et al., 2019). The variables of body height and mass aligned with studies on professional soccer players in Belgium and Serbia (Colosio et al., 2020; Katanic et al., 2023). However, it should be noted that professional soccer players in this study had an average body fat percentage of 9.55%, which is slightly lower than the average values for elite soccer players, ranging between 9.9% and 11.9% (Slimani and Nikolaidis, 2017). It is well known that anthropometric characteristics can provide a general advantage in matches, which is a positive predictor of the outcome of the game (Ré et al., 2014). Our results are inconsistent with some studies which suggest that anthropometric variables do not predict match performance and should be used with caution (Ré et al., 2014; Rienzi et al., 2000). The regression analysis in our study showed that body composition variables such as muscle and fat content were associated with jogging, high-speed running, and total distance covered. This is in line with the study by Radzimiński et al. (2019) who found that professional soccer players with lower body fat mass covered greater total distances and performed more high-intensity sprints. Similar results were also reported by Savolainen et al. (2023) who showed that the body fat percentage was related to running and high-speed running performance. Additionally, this study found a relationship between body mass and the BMI with the mentioned movement performance, while lean body mass did not affect MRP. Also, some research discovered negative correlations between subscapular skinfold and overall distance, high-intensity running, and sprinting in youth athletes (Sporiš et al., 2017). This all emphasizes the significant role of body composition in professional soccer. Although optimal muscle mass is desirable and has shown an effect on certain MRP variables in our study, body fat content is a more significant variable and should be lower in professional soccer players (Cug et al., 2024). The mechanisms behind this fact relate to the excess fat mass in a soccer player's body acting as ballast, further burdening the player in their movements throughout the game, and thus negatively affecting the expression of their performance. Body composition, particularly muscle mass and fat content, influences MRP due to its impact on metabolic efficiency, movement optimization, and overall fatigue resistance. It is known that higher muscle mass positively correlates with force production, which is essential for realization of biomechanical patterns fundamental in soccer. Furthermore, muscle mass also supports endurance by improving metabolic efficiency and delaying fatigue (Enoka and Duchateau, 2008). On the other hand, excess body fat increases the energy cost of movement in general and reduces overall performance efficiency.

When analyzing motor performance, our players had a slightly weaker score at 10 m compared to Portuguese players (1.57 s; Barrera et al., 2023), but better results than players from the English Premier League and the Spanish third division (1.70–1.72 s; Northeast et al., 2019; Papla et al., 2020). Our players had a similar time (2.92 s) at the 20-m sprint compared to Premier League players (2.94 s) and better times than players from the Portuguese first division (2.99 s; Barrera et al., 2023) and Polish second-division players (3.02 s; Papla et al., 2020). At 30 m, our players achieved a better time of 4.05 s than first-division players from Portugal (4.55 s; Barrera et al., 2023).

Considering motor abilities, an association was found between sprint speed variables at 10–30 m, the 10/20 acceleration index, as well as explosive abilities (CMJ) and jogging and total distance covered, while SJ performance was only connected with jogging. Furthermore, sprinting performance (10 m, 20 m, 30 m) and explosive power (CMJ, SJ) were associated to MRP, most likely being a reflection of players’ fundamental motor abilities, i.e., acceleration, deceleration and agility observed in real-world competitive scenarios. Sprint times significantly positively correlate with neuromuscular coordination and Type II fast-twitch muscle fibers recruitment, which are key factors for high-speed running and quick bursts of activity during matches (Ross et al., 2001). Jump performance (CMJ, SJ) reflects lower limb strength and power, which are essential for quick accelerations, decelerations, and repeated high-intensity efforts (Buchheit et al., 2010). Our results are in line with those of Savolainen et al. (2023) who found an association between the 30-m sprint and high-speed running performance. Goncalves et al. (2021) established a connection between linear sprints and the number of accelerations, decelerations, and sprints during matches. In the same study, the authors (Goncalves et al., 2021) found a connection between jumps and high-intensity accelerations during the game, as well as a link between agility and running performance during matches, which was not observed in our study.

With regard to explosive strength variables in jump measurements, our soccer players achieved significantly better results (SJ: 42.00 cm and CMJ: 53.44 cm) compared to elite Portuguese players (SJ: 36.0 cm and CMJ: 37.2 cm; Barrera et al., 2023) and Italian players (SJ: 37.6 cm and CMJ: 40.5 cm; Castagna and Castellini, 2013). Although it is difficult to compare other agility variables with those of other soccer players due to the specificity of the tests, speed indices and explosive strength abilities suggest that our sample represented elite soccer players who, in most cases, achieved slightly better results than their counterparts from other countries. Additionally, in terms of endurance, in the shuttle run test, our players covered an average distance of 2476.59 m, which was significantly more than the 1,658.9 m achieved by semi-professional soccer players from Greece (Nassis et al., 2010), further supporting the fact that this was a representative elite sample of soccer players.

In terms of movement performance, the total distance covered was 10,799 m. It can be observed that the results of the MRP variables in our study align with previous research on elite Spanish (Ponce-Bordón et al., 2024) and Croatian soccer players (Modric et al., 2019). These values correspond to general reference standards for elite soccer players, which range from 9 to 13 km (Gomez-Piqueras et al., 2019). While our players covered an average of 611 m in high-intensity running, which is slightly lower than players from the Croatian national team (617–661 m; Jerkovic et al., 2022), they covered 488 m in high-speed running, which corresponds to the values of players in the Croatian First League, depending on their playing position (460–499 m, Jerkovic et al., 2022).

The nature of soccer is characterized by a combination of low-, high-, and moderate-intensity actions during the game, which highlights the importance of endurance capacities (Jastrzębski et al., 2025). In this regard, the aerobic shuttle-run test showed a connection with high-speed running, which corresponds with previous research (Krustrup et al., 2003; Rampinini et al., 2006). These findings also align with studies that have found a link between cardiorespiratory variables and movement performance (Radaković et al., 2024b; Radzimiński et al., 2019), while in the study by Savolainen et al. (2023), it was even found that VO_2max_ was related to all movement performance variables. These results emphasize the importance of developing the soccer's aerobic energy system which provides about 90% of the energy during a soccer match (Tomlin and Wenger, 2001). Therefore, soccer players with a more efficient aerobic system can maintain higher intensity before fatigue sets in, allowing them to cover greater distances in high-intensity running. Overall, our results emphasize the importance of physical preparation and the optimal level of motor abilities in soccer players, highlighting their impact on total match running performance (Radaković et al., 2024b; Rampinini, 2006). However, it should be noted that the connection between motor abilities and high-speed performance was absent, and the reasons for this absence may lie in insufficiently specific tests, as well as the small sample size of participants and analyzed matches.

The main strength of the study is that it is the first to investigate the combined influence of both anthropometric characteristics and motor abilities on movement performance. Additional strength of the study lies in the fact that it is the first research conducted with professional soccer players as a relatively big and representative sample while comprehensive testing of anthropometric characteristics, motor abilities, and movement performance was conducted.

## Limitations

One of the main limitations of the research is that players were not categorized according to positions or team lines. From a methodological perspective, it is not possible to generalize the results because of a relatively small sample size of participants compared to a large number of predictor variables. Moreover, only one match was assessed for each soccer player, whilst multiple matches would be needed to obtain more accurate insights into the players' movement patterns. Additionally, while BIA methods are considered valid, they may have certain limitations when applied to athletes, and future studies should consider using the DXA method. Also, future studies should investigate this topic by including players from different leagues, as well as soccer players of various competitive levels.

## Conclusions

In conclusion, this study highlights the significant impact of both anthropometric characteristics and motor abilities on match running performance (MRP) in elite soccer players. The findings emphasize the effects of body composition, particularly muscle and fat content, jogging, high-speed running, and total distance covered. Additionally, sprinting performance, agility, and explosive strength, as well as endurance capacity, were found to be key predictors of various MRP variables. The results offer valuable insights into the interplay between morphological and motor factors, providing a foundation for the development of targeted training programs aimed at optimizing MRP in elite soccer players. These findings underscore the importance of considering both physical and physiological variables when designing training regimens to enhance performance on the field.
